# Exosomes as renal inductive signals in health and disease, and their application as diagnostic markers and therapeutic agents

**DOI:** 10.3389/fcell.2015.00065

**Published:** 2015-10-20

**Authors:** Mirja Krause, Anatoliy Samoylenko, Seppo J. Vainio

**Affiliations:** Biocenter Oulu, Infotech Oulu, Developmental Biology Lab, Faculty of Biochemistry and Molecular Medicine, Center for Cell Matrix Research, University of OuluOulu, Finland

**Keywords:** extracellular vesicles, exosomes, kidney development, diagnostic markers, therapeutics, renal disease, renal cancer

## Abstract

Cells secrete around 30–1000 nm membrane-enclosed vesicles, of which members of the subgroup between 30 and 100 nm are termed exosomes (EXs). EXs are released into the extracellular space and are widely present in body fluids and incorporated mRNA, miRNA, proteins, and signaling molecules. Increasing amounts of evidence suggest that EXs play an important role not only in cell-to-cell communication but also in various physiological and disease processes. EXs secreted by kidney cells control nephron function and are involved in kidney diseases and cancers. This makes them potential targets for diagnostic and therapeutic applications such as non-invasive biomarkers and cell-free vaccines and for use as drug delivery vehicles. This review provides an overview on the known roles of EXs in kidney development and diseases, including renal cancer. Additionally, it covers recent findings on their significance as diagnostic markers and on therapeutic applications to renal diseases and cancers. The intention is to promote an awareness of how many questions still remain open but are certainly worth investigating.

## Introduction

Cells from various organisms, including all the eukaryotes and many prokaryotes, release extracellular vesicles of different types into their environment. The term exosomes (EXs) describes those vesicles which are of endosomal origin. They are small (30–100 nm) membrane-enclosed vesicles which are secreted by various cell types, and can be found in biological fluids such as blood, semen, saliva, and urine (reviewed by Raposo and Stoorvogel, 2013; Yanez-Mo et al., 2015). Initially these were considered to take part in the cell's waste management but evidence has accumulated that they are instrumental in intercellular and system (humoral)-level communication in organisms. The striking property of EXs is that they can transfer important compounds such as membrane and cytosolic proteins, lipids, mRNA, miRNA (Valadi et al., 2007), and even DNA between cells. Thus, they provide a novel but apparently evolutionary ancient platform for cell-to-cell and tissue interactions (Valadi et al., 2007).

Even though the putative developmental role of EXs remains in most respects unclear, it is emerging that at least many of the key developmental signaling pathways are coordinated by EXs (Raposo and Stoorvogel, 2013; Urbanelli et al., 2013). Kidney cells, for instance, appear to release vesicles presumably in a developmentally regulated manner, and the currently available data suggest that EXs may initiate and regulate organogenesis. In addition, they may also take part in processes such as regeneration and the development of diseases such as cancer. Interestingly, kidney-derived vesicles may also have an impact on the cells of certain other organs (Grange et al., 2011) and even the immune system. The aim of this review is to summarize the roles that have been demonstrated for EXs in kidney development and disease, especially in kidney tumorigenesis.

## Exosomes as renal inductive signals, diagnostic markers, disease, and therapeutic agents

### Biogenesis and signal transduction

**Figure 1:**
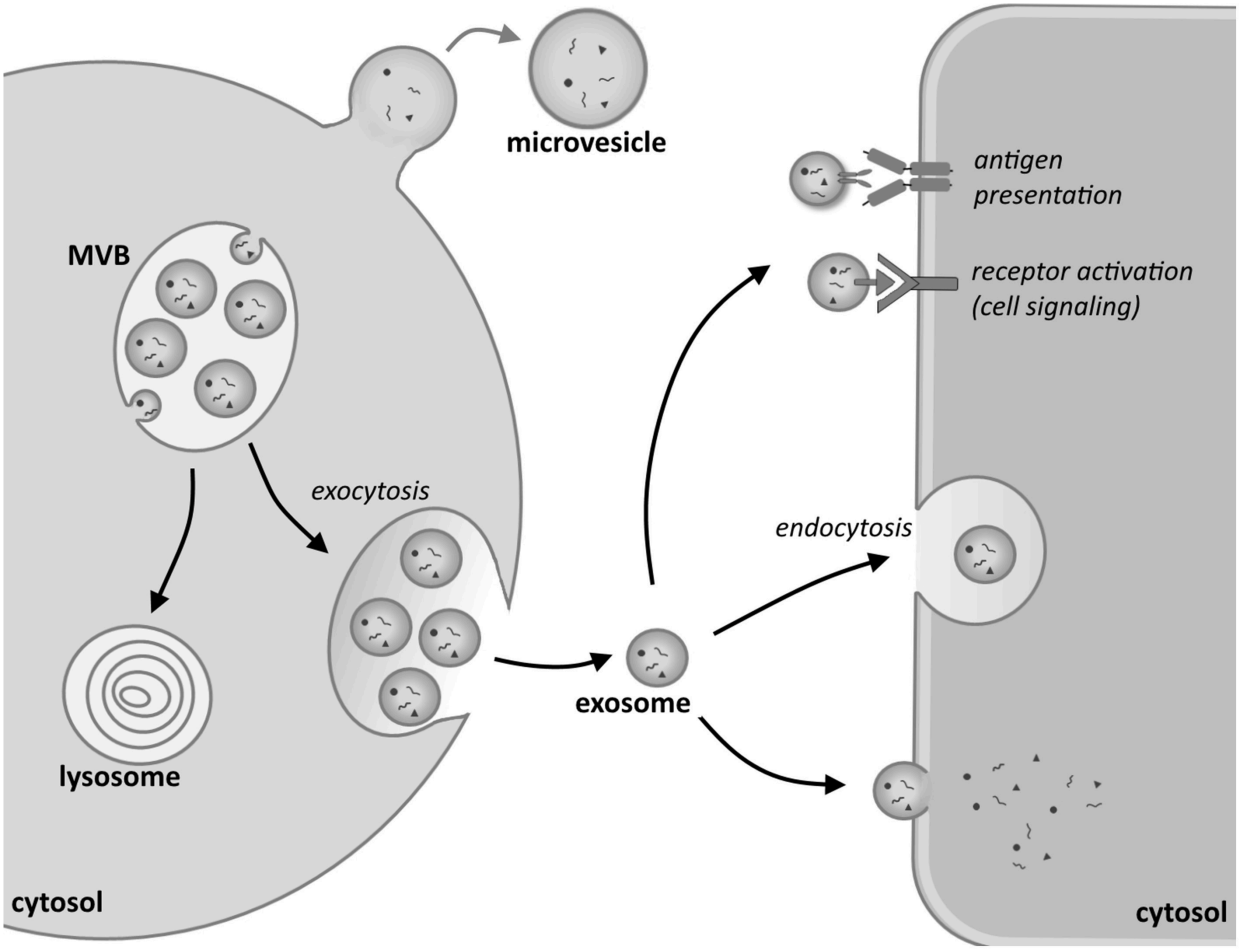
**Biogenesis and uptake of exosomes**. Inward budding of the membrane of an endosomal vesicle in the cytosol of the donor cell leads to the formation of multivesicular bodies (MVBs). These can either turn into lysosomes, whereupon their content will be degraded, or fuse with the plasma membrane, releasing their content into the extracellular environment. These vesicles are called exosomes. By contrast, larger microvesicles are formed when the cell membrane directly buds outwards, giving rise to micelles. Exosomes can interact with recipient cells in various ways. Signaling can be initiated by an antigen-antibody interaction in the recipient cell or by the activation of a receptor on the cell membrane of the target cell. Exosomes can also be taken up by endocytosis or fuse directly with the plasma membrane. Upon fusion their cargo is released into the cytosol of the target cell.

The biogenesis of EXs is a complex intracellular process that forms part of the endosomal cell sorting machinery, in which an array of regulatory factors has been identified (Stoorvogel et al., 2002; Théry et al., 2002; Kowal et al., 2014). Upon the initiation of EX assembly an early endosome is formed by an inward budding of the cellular membranes via endocytosis. In association with this budding the endosomal membrane undergoes a second inward folding to generate further smaller vesicles inside the endosomal lumen. The process is collectively called the formation of multivesicular bodies (MVB; Figure 1).

The machinery known as the Endosomal Sorting Complex Required for Transport (ESCRT) is involved in the formation of MVBs, although it also entails an ESCRT-independent mechanism involving proteins such as tetraspanins and lipids (for details, see recent reviews by Hanson and Cashikar, 2012; Henne et al., 2013; Colombo et al., 2014; Kowal et al., 2014). The assembled MVBs can then fuse either with lysosomes, leading to their degradation, or alternatively with the cellular plasma membrane (PM), which releases their vesicles, called from here on exosomes (EXs), into the extracellular milieu of the cell. The fusion of MVBs with the PM was first described in the hematopoietic system, namely in differentiating red blood cells (Pan and Johnstone, 1983), and thereafter in several other cell types such as B-cells, T-cells, dendritic cells (Escola et al., 1998; Zitvogel et al., 1998; Blanchard et al., 2002), mast cells (Raposo et al., 1997), and yeast cells (Henne et al., 2013). The biogenesis of EXs has also been described in epithelial cells, including those of the kidney (Knepper and Pisitkun, 2007), being the cells that line the renal tubule lumen (Pisitkun et al., 2004).

On their release EXs interact with a recipient cell in several ways. A schematic overview of the biogenesis of EXs and how they enter target cells and interact with them is depicted in Figure 1. As one mode of interaction, EXs bind to the target cell via membrane receptors such as the Major histocompatibility complex (MHC) that mediates binding to T-cells (Denzer et al., 2000; Nolte-'t Hoen et al., 2009). Another way in which EXs bind is via recognition of the ligands on their lipid bilayer by means of specific cell surface receptors. Indeed, many common ligand/receptor pairs such as recognition by integrins or tetraspanins have been identified. The capacity of an EX to bind to a given cell depends to a great deal, however, on the cellular content, and also the characteristics of the donor cell from which the EXs originated. The most prominent binding mechanisms have been reviewed recently by Colombo et al. (2014) and are therefore, not reviewed here. Besides receptor-mediated binding, the entry of EXs into a target cell may involve endocytosis (Morelli et al., 2004), phagocytosis (Feng et al., 2010; Christianson et al., 2013; Yanez-Mo et al., 2015), or pinocytosis (Parolini et al., 2009; Théry et al., 2009; Yanez-Mo et al., 2015). These processes can also be receptor-mediated, aided by several other proteins such as dynamin (Fitzner et al., 2011) and clathrin (Mulcahy et al., 2014; Tian et al., 2014), which regulate entry. In the case of kidney-derived cells such as COS-7, the cells seem to internalize EX through lipid raft-mediated endocytosis which is negatively regulated by caveolin-1 (Svensson et al., 2013). It is currently poorly understood whether EX uptake mechanisms are common or specific to each cell type (Gildea et al., 2014). Furthermore, there is also some debate as to how widely certain EXs can influence cell behavior (White et al., 2006; Svensson et al., 2013).

### Exosomes in kidney physiology

The currently available data suggest that the extracellular vesicles are coupled to normal development and various diseases. Given that EXs contain key regulatory signals such as mRNA and miRNA and can transfer them from one cell type to another, they may have a profound influence on target cell homeostasis (Montecalvo et al., 2012; Stoorvogel, 2012; Tomasoni et al., 2012; Zhang et al., 2015b). The capacity of EXs to transport miRNA is of particular importance when considering participation in developmental control in general (Tang et al., 2007). Even though it still remains unknown whether EXs indeed play a crucial role in morphogenesis, the current results suggest that at least urinary EXs, besides being involved in the secretion of senescent proteins as exocytic waste (Pisitkun et al., 2004; Knepper and Pisitkun, 2007) may also have other roles (van Balkom et al., 2011; Fang et al., 2013). In the light of current data, the systemic serum containing EXs cannot cross the kidney filter within the glomerulus under normal conditions (Pisitkun et al., 2004; Gildea et al., 2014), but whether this changes in the presence of kidney anomalies remains open.

Proteomic studies of urinary EXs have identified proteins that characterize certain nephron segments such as the glomerulus and Henle's loop. A summary is given in Table 1 (Pisitkun et al., 2004; Miranda et al., 2010; Dear et al., 2013). The available data suggest that most, if not all, of the nephron cell types have the capacity to secrete vesicles. EXs may play an important role in mediating cell-to-cell communication along the nephron with potential significance for kidney performance, and it has been shown that the function of the EXs within the nephron may be the adaptation of nephron function to changes in physiology, i.e., in homeostasis. This possibility is based on the observation that different segments of the nephron secrete and take up EXs differentially (Dimov et al., 2009). Renal EXs can also transfer functional molecules such as aquaporin-2 between cells (Street et al., 2011), although their uptake capacity becomes restricted in the fully matured adult kidney. This is probably caused by accumulation of the tubular Tamm-Horsfall protein, which prevents contacts between EXs and the tubular luminal cells unless this protein is degraded locally (van Balkom et al., 2011). Hence, the luminal epithelial cells of the nephron apparently secrete the EXs found in urine. Furthermore, proteins which are specifically associated with the urinary bladder and prostate gland have also been found in urine (Musante et al., 2014), although further studies are needed to provide conclusive evidence of this.

**Table 1:** **Proteins found in human urinary exosomes that are specific to or enriched in given regions of the kidney**.

**Kidney region**	**Gene symbol**	**Full gene name**	**Species**	**Exosome sample**	**Identified molecule**	**Associated diseases**
Glomerulus	PODXL	Podocalyxin-like Protein	Homo sapiens	Urine	Protein	Diabetic nephropathy
	NPHS2	Podocin	Homo sapiens	Urine	mRNA	Focal segmental glomerulosclerosis, nephrotic syndrome [MIM: 600995]
	LGALS1	Galectin-1	Homo sapiens	Urine	mRNA	–
	HSPG2	Heparan Sulfate Proteoglycan 2	Homo sapiens	Urine	mRNA	Schwartz-Jampel syndrome type 1 [MIM: 255800]
1st convoluted tubule	gp330 precursor	Glycoprotein 330 Precursor	Homo sapiens	Urine	Protein	Renal aminoglycoside accumulation and nephrotoxicity, Donnai-Barrow syndrome
	CUBN	Cubilin (Intrinsic Factor-Cobalamin Receptor)	Homo sapiens	Urine	Protein, mRNA	Megaloblastic anemia 1 [MIM: 261100]
	AQP1	Aquaporin1	Homo sapiens	Urine	protein, mRNA	Nephrogenic diabetes insipidus, Aquaporin 1 deficiency, Colton-Null [MIM: 110450]
	LRP2	Megalin	Homo sapiens	Urine	mRNA	Heymann nephritis, proteinuria
	CA4	Carbonic Anhydrase 4	Homo sapiens	Urine	mRNA	Diabetic nephropathy, Proximal renal tubular acidosis [MIM: 114760]
	ANPEP	Alanyl Aminopeptidase	Homo sapiens	Urine	protein	Hypertension [MIM: 151530]
	NAPSA	NapsinA	Homo sapiens	Urine	Protein	Kidney carcinoma, renal neoplasms
	CLCN 5	Chloride Channel Protein 5	Homo sapiens	Urine	mRNA	Dent's disease
	GGT1	γ-glutamyltransferase	Homo sapiens (male)	Urine	Protein	–
	APN	Aminopeptidase N	Homo sapiens (male)	Urine	Protein	–
Henle's loop	AQP1	Aquaporin 1	Homo sapiens	Urine	Protein	Nephrogenic diabetes insipidus, Aquaporin 1 deficiency, Colton-Null [MIM: 110450]
	UMOD	Uromodulin	Homo sapiens	Urine	Protein	Hyperuricemic nephropathy, Medullary cystic kidney disease-2 (MCKD2) [MIM: 603860], familial juvenile hyperuricemic nephropathy (FJHN) [MIM: 16200]
	THP	Tamm-Horsfall Protein	Homo sapiens (male)	Urine	Protein	Mckd2 [mim: 603860], fjhn [mim: 16200]
	CD9	Cluster of Differentiation (Tetraspanin)	Homo sapiens (male)	Urine	Protein	–
	BDKRB1	Bradykinin B1 Receptor	Homo sapiens	Urine	mRNA	–
	CALCR	Calcitonin Receptor	Homo sapiens	Urine	mRNA	Kidney stone disease
	SCNN1D	Amiloride-sensitive Sodium Channel Subunit Delta	Homo sapiens	Urine	mRNA	–
2nd convoluted tubule	SLC12A3 (NCC)	Thiazide-sensitive Na-Cl Cotransporter	Homo sapiens	Urine	Protein	Gitelman syndrome [MIM: 263800]
Collecting ducts	AQP2	Aquaporin 2	Homo sapiens	Urine	mRNA	Nephrogenic diabetes insipidus type 1 [MIM: 222000] [MIM: 125800]
	ATP6V1B1	V-ATPase B1 Subunit	Homo sapiens	Urine	mRNA	Distal renal tubular acidosis [MIM: 267300]
	SLC12A1	Kidney-specific Na-K-Cl Symporter	Homo sapiens	Urine	mRNA	Bartter-Syndrome type 1, 2, 3 [MIM: 601678, 241200, 607364]
	MUC1	Mucin-1	Homo sapiens (male)	Urine	Protein	Renal cell carcinoma, Medullary cystic kidney disease type 1 (MCKD1) [MIM:174000]
	RHCG	Rh type C glycoprotein	Homo sapiens (male)	Urine	Protein	–

*Data compiled from Pisitkun et al. (2004), Gonzales et al. (2009), Miranda et al. (2010), Dear et al. (2013), and Musante et al. (2014) and the Online Mendelian. Inheritance in Man OMIM® website (http://www.omim.org/, see MIM numbers for reference)*.

When considering the embryonic kidney and the potential of EXs for taking part in its developmental programming, many of the proteins known to control organogenesis, including growth factors (see the recent review by Krause et al., 2015) are in fact found in the EXs that have been characterized from a variety of cell lines (Table 2). The Wnt-family members and their signal transduction pathway are critical for kidney development, and interestingly, several Wnt proteins and their downstream factors such as β-catenin are associated with EXs and can also mediate activation of the pathway (Table 2; Zhang et al., 2015a). It can be speculated that a panel of key developmental signals may also be associated with and transported to the target cells via EXs during kidney development.

**Table 2:** **Genes of importance during kidney development found in exosomes of various origins**.

**Gene symbol**	**Full gene name**	**Species**	**Exosome sample**	**Identified molecule**	**References**	**ExoCarta ID (Mathivanan and Simpson, 2009)**
Wnt4	Wingless-type MMTV Integration Site Family, Member 4	Homo sapiens	Umbelical cord mesenchymal stem cells	protein	Zhang et al., 2015a	No
Wnt11	Wingless-type MMTV Integration Site Family, Member 11	Mus musculus	Mast cells	mRNA	Valadi et al., 2007	ExoCarta_22411
Notch2	Neurogenic locus notch homolog protein 2	Homo sapiens	Ovarian cancer cells	Protein	Liang et al., 2013	ExoCarta_4853
		Homo sapiens	Colorectal cancer cells	Protein	Demory Beckler et al., 2013	ExoCarta_4853
		Bos taurus	Milk	Protein	Reinhardt et al., 2013	ExoCarta_513730
BMP4	Bone Morphogenetic Protein 4	Homo sapiens	Colorectal cancer cells	Protein	Demory Beckler et al., 2013	ExoCarta_652
FGFR1	Fibroblast Growth Factor receptor 1	Homo sapiens	Ovarian cancer cells	Protein	Liang et al., 2013	ExoCarta_2260
OSR1	Oxidative Stress responsive 1	Homo sapiens	Ovarian cancer cells	Protein	Liang et al., 2013	ExoCarta_9943
		Homo sapiens	Thymus	Protein	Skogberg et al., 2013	No
		Homo sapiens	Urine	Protein	Gonzales et al., 2009	No
		Rattus norwegicus	Reticulocytes	Protein	Carayon et al., 2011	ExoCarta_316064
WT1	Wilms Tumor 1 Homolog	Mus musculus	Mast cells	mRNA	Valadi et al., 2007	ExoCarta_22431
					Ranghino et al., 2014	No
β-catenin	Cadherin-associated protein beta	Mus musculus	Dendritic cells	Protein	Chairoungdua et al., 2010	No

If this is the case, then EXs constitute a new critical mechanism in the control of kidney development by transferring and integrating key inductive signals. Thus, their presence during kidney development should be explored further: Which cells secrete EXs under which environmental conditions, and do these EXs populations differ one from another? Further analysis should then reveal details of their content, transport mechanisms and physiological roles during kidney development.

The putative role of EXs has recently been explored in Madin-Darby canine kidney cells (MDCK; Kwon et al., 2014). When these cells are subjected to the hepatocyte growth factor (HGF) their proliferation is stimulated and eventually tubular cysts form. Changes in the expression of a specific G protein-coupled receptor, GPRC5B, are associated with EXs being secreted by these cells. This protein is also up-regulated in tubulogenesis, while the exosomal delivery of GPRC5B induces extracellular signal-regulated kinase 1/2 (Erk1/2). As the GPRC5B is expressed in the ureteric bud of the embryonic kidney, this may suggest that it also plays a role in organogenesis.

### The role of exosomes in kidney regeneration and diseases

While the role of kidney-derived EXs in physiological processes remains poorly investigated, EXs have been found to exercise beneficial or adverse functions in the development of several kidney diseases (Borges et al., 2013a; Fang et al., 2013). For example, vesicles derived from mesenchyme stem cells (MSCs) or endothelial stem cells can promote kidney regeneration (Borges et al., 2013a). The positive impact of MSCs on both acute and chronic kidney injury (AKI and CKI) was first attributed to their role in directly replacing renal tubular cells, but later it became clear that these cells rather provide paracrine support for endogenous regeneration (Biancone et al., 2012). At present the role of MSCs has been assigned in part to the secretion of EXs (Camussi et al., 2010; Biancone et al., 2012; Borges et al., 2013a), e.g., in that MSC-derived EXs can enhance regeneration of the rat kidney epithelium when injured by ischemia-reperfusion (Gatti et al., 2011). This EX-mediated recovery involves cell-to-cell transfer of mRNAs and/or microRNAs and may be connected with the inhibition of renal cell apoptosis and the stimulation of tubular epithelial cell proliferation. Moreover, by reducing the acute injury, the EXs also protected the rats from later CKI development 6 months after the operation (Gatti et al., 2011).

Other studies have reported a regenerative potential in microvesicles (MVs) produced by bone marrow MSCs in glycerol-induced (Bruno et al., 2009), cisplatin-induced (Bruno et al., 2012), and gentamicin-induced (Reis et al., 2012) AKI models *via* a mechanism dependent on RNA delivery. Here the protective effect of EXs was mainly ascribed to an increase in surviving tubular cell proliferation (Bruno et al., 2009; Reis et al., 2012) and a decrease in tubular epithelial cell apoptosis (Bruno et al., 2009, 2012; Reis et al., 2012). These effects are thought to occur via the up-regulation of anti-apoptotic genes and down-regulation of genes involved in the execution phase of cell apoptosis (Bruno et al., 2012).

It has also been found that the development of chronic tubular injury is inhibited by multiple injections of MVs, while the effect of a single injection was not sufficient to prevent CKI (Bruno et al., 2012). Like bone marrow MSC-derived EXs, human umbilical cord MSC-derived EXs also demonstrated a protective effect on cisplatin-induced nephrotoxicity *in vivo* and *in vitro*, whereas human lung fibroblast-derived EXs did not (Dorronsoro and Robbins, 2013; Zhou et al., 2013a). Zhou et al. (2013a) demonstrated that these EXs can reduce cisplatin-mediated renal oxidative stress and apoptosis in rats *in vivo* and increase the proliferation of renal tubular epithelial NRK-52E cells in culture. They also showed that human umbilical cord MSC-derived EXs can reduce Bax (bcl-2-like protein 4) level and increase Bcl-2 (B-cell lymphoma 2) in order to inhibit apoptosis and stimulate Erk1/2, thereby increasing proliferation after cisplatin-induced injury in the kidney. Another group has shown that EXs isolated from peripheral blood-derived endothelial progenitor cells can prevent AKI in an ischemia-reperfusion rat model (Cantaluppi et al., 2012). In this case the miRNAs that modulate proliferation, angiogenesis and apoptosis were found to be responsible for the protective effects of EXs.

Kidney epithelial cells are another source of the EXs involved in kidney regeneration. It was found that administration of the epithelium-derived exosomal ATF3 (activating transcription factor 3) mRNA attenuates ischemia/reperfusion-induced kidney injury by inhibiting monocyte chemotactic protein-1 (MCP-1)-induced macrophage infiltration (Chen et al., 2014b). While all the above-mentioned studies demonstrate beneficial effects of EXs on kidney regeneration, it was found on the other hand that EXs produced by injured proximal tubular epithelial cells in a murine model of hypoxic kidney fibrosis after unilateral ureteral obstruction can initiate tissue repair/regenerative responses and activate fibroblasts, leading to fibrosis (Borges et al., 2013b). Fibroblast proliferation and the production of matrix proteins were particularly dependent on EXs delivering TGF-β1 (transforming growth factor β1) mRNA (Borges et al., 2013b).

Interestingly, certain *in vitro* studies published more than 20 years ago demonstrated that renal brush border-derived MVs about 100 nm in diameter can induce and promote calcium oxalate crystallization (Nagasawa et al., 1992; Anderson et al., 2010), which is one of the features of nephrolithiasis (kidney stone formation), a pathological kidney condition leading to fibrosis and chronic renal failure (Anderson et al., 2010).

Since, the endothelial cells are connected to the control of blood flow, pressure and clotting, they are prime targets when considering the development of EX-based therapies, and also the treating of renal diseases (van Balkom et al., 2011). It has been shown that circulating levels of endothelial-derived MVs are significantly higher in chronic renal failure patients than in healthy subjects (Faure et al., 2006). Moreover, blood levels of these MVs correlate with endothelial dysfunction and arterial stiffness in end-stage renal failure hemodialysis patients (Amabile et al., 2005). In contrast to endothelium-derived MVs, those produced by platelets or erythrocytes do not seem to be connected with endothelial dysfunction (Amabile et al., 2005). In another study it was found that the number of endothelial-derived microparticles was inversely correlated with brachial artery and aortic laminar shear stress values in end-stage renal disease patients with a high cardiovascular risk (Boulanger et al., 2007).

It may be assumed that the action of cell-damaging agents such as low shear stress and increased arterial stiffness contributes to endothelial apoptosis through a substantial release of endothelial MVs (Amabile et al., 2005; Boulanger et al., 2007). The pathogenetic effects of EXs and other circulating microparticles in promoting vascular calcification and sclerosis in chronic kidney disease (CKD) are nevertheless not yet clearly defined (Anderson et al., 2010; Fang et al., 2013). Rather surprisingly, Neal et al. (2011) found that the levels of circulating miRNAs in patients with different stages of chronic kidney failure, including those receiving hemodialysis treatment, were reduced by comparison with patients having mild renal impairment or normal renal function. This observation might be explained by the fact that many circulating miRNAs are bound to Argonaute 2-containing protein complexes (Arroyo et al., 2011) or to high-density lipoproteins (Vickers et al., 2011) rather than within EXs. It has been demonstrated in a rat model of CKD induced by 5/6 nephrectomy that the administration of conditioned medium from embryonic MSC has a therapeutic effect, whereas MSC-derived EXs tested in the same experimental setting showed no protective effect on the kidney (van Koppen et al., 2012).

### Exosomes in kidney cancer

Although tumor cells secret large amounts of various MVs that enter the blood and other body fluids (Lee et al., 2011; Azmi et al., 2013), the isolation of cancer EXs from patients remains a serious problem due to the lack of specific markers that can distinguish cancer-derived from non-cancer-derived EXs. One such marker that has been identified recently is glypican-1, detected on EXs derived from the serum of patients with pancreatic cancer but not on EXs from healthy subjects or subjects with chronic pancreatitis (Melo et al., 2015). Various biological roles have been proposed for EXs in cancer, such as the expulsion of key proteins and miRNA from cells, the removal of anti-cancer drugs and the release of signaling and regulatory molecules (Lee et al., 2011; Azmi et al., 2013). The properties of EXs may also enable them to take part in the control of cell proliferation, cell survival, angiogenesis, metastasis and immune response (Azmi et al., 2013; Fang et al., 2013). EX-released factors promote stromal remodeling and hypoxia-mediated epithelial mesenchyme transformation, which is critical for the evolution of cancer (Nieto, 2011). EXs can also stimulate the proliferation of fibroblasts by causing a desmoplastic reaction, and they can induce immune escape mechanisms by suppressing antigen-specific immune responses and by up-regulating immunosuppressive cell differentiation and the functioning of these cells (Azmi et al., 2013; Minciacchi et al., 2015). For these reasons EXs form a critical aspect of tumorigenesis.

Much less is known at present about the roles of EXs in kidney tumorigenesis, however. The relevant findings are that the vesicles released by renal carcinoma stem cells (rCSCs) derived from a tumor-bearing patient can trigger angiogenesis and promote lung metastasis when studied with the SCID (immunocompromised severe combined immunodeficient) mouse model (Grange et al., 2011). Grange et al. (2011) showed that rCSCs are secreted by a subset of tumor-initiating cells characteristically expressing the mesenchymal stem cell marker CD105, and that EXs derived from these rCSCs were able to stimulate the growth and invasiveness of normal HUVEC (human umbilical vein endothelial cells). In addition, these EXs increased the formation of capillary-like structures in culture and in induced vessel formation when cells treated with them were grafted into SCID mice.

More detailed molecular characterization of the CD105-positive EXs pointed to significant differences in mRNA and miRNA content as compared with EXs that were negative for CD105. Consistent with their properties, the rCSC-derived EXs have mRNAs that encode for proangiogenic factors such as vascular endothelial growth factor (VEGF), fibroblast growth factor (FGF), angiopoietin-1, ephrin A3, and matrix metalloproteinases (MMP)-2 and -9. Similarly, these were reported to be absent from the CD105-negative tumor cell-derived EXs. In addition to these findings, miRNAs that have been reported to be involved in biological processes such as the control of transcription, cell adhesion and cell proliferation were enriched in CD105-positive EXs (Grange et al., 2011, 2014).

Another indication that EXs derived from kidney cancer cells may be involved in the cancerous state and its severity, including the formation of metastases, came from the work of Tauro et al. (2013), who performed a proteomic analysis by comparing EXs released by normal and oncogenic H-Ras (21D1) transfected MDCK cells. While the control and 21D1-MDCK cell-derived EXs were similar in their morphology, the 21D1-derived EXs had high levels of proteases, annexins, integrins, and other secreted proteins which are typically associated with the formation of premetastatic niches. The ability of primary cancer to make changes in normal tissue located in a pre-metastatic niche prior to the arrival of metastasizing cells is an important feature that facilitates sustained cancer growth (Wels et al., 2008).

The role of EXs in the “education” of normal cells toward a pro-metastatic phenotype has been demonstrated not only for renal cell carcinoma but also for other cancers, including melanomas (Peinado et al., 2012) and breast cancer (Fong et al., 2015). Elsewhere, the transfection of MDCK cells with oncogenic H-Ras has been found to induce the release of EXs that contain factors known to control nuclear assembly, transcription, splicing, and translation. The most abundant protein in the 21D1-derived EXs was the Y-box-binding protein (YBX1), which is a DNA and RNA- binding transcription factor involved in DNA replication, DNA repair, transcription, pre-mRNA splicing, and mRNA translation (Eliseeva et al., 2011).

Several studies have been conducted employing 786-0 human renal adenocarcinoma cells and targeted EXs in carcinogenesis (Zhang et al., 2013; Chen et al., 2014a; Du et al., 2014). Like the rCSC-derived EXs (Grange et al., 2011) the 786-0-derived ones promote tubulogenesis in HUVECs (Human umbilical vein endothelial cells; Zhang et al., 2013), and it has been speculated that the pro-angiogenic effect of these renal EXs may be mediated via down-regulation of hepaCAM (hepatic and glial cell adhesion molecule), a hepatocyte cell adhesion molecule, and up-regulation of VEGF. In turn, it has been shown that the EX-mediated down-regulation of hepaCAM expression is in effect mediated by Akt phosphorylation connected with the enhanced renal carcinoma cell proliferation (Jiang et al., 2014). Collectively, the 786-0-derived EXs enhance cell migration, invasion and chemokine receptor type 4 (CXCR4) and MMP-9 expression and concurrently reduce the adhesion of 786-0 cells (Chen et al., 2014a).

A comprehensive report on the influence of EXs on renal carcinoma 786-0 cells has been published recently by Du et al. (2014), who investigated the putative effects of EXs released by human Wharton's jelly mesenchyme stem cells (hWJ-MSCs). These EXs promoted cell proliferation, cell migration, and progression of the cell cycle from G0/G1 to the S phase and the HGF/c-Met, Akt, and Erk1/2 pathways in these cells. Moreover, the hWJ-MSCs-derived EXs stimulated tumorigenesis in the 786-0-cells and also enhanced tumor size. At the molecular level, the EXs induced cyclin D1, MMP-2, and MMP-9 expression in a BALB/c nude mouse model (Du et al., 2014). Meanwhile, RNase pre-treatment abrogated these exosomal effects, indicating that the RNA delivered via EXs serves as a crucial mediator (Du et al., 2014).

Little is known as to whether the cells in various organs take up the cancer-derived EXs selectively. Rana et al. (2012) found that small differences in the EX-tetraspanin complexes that originated from different rat tumor cell lines greatly influenced the cell type to which the EXs were targeted *in vitro* or *in vivo*. When tetraspanin-8+ EXs were monitored after 24 h of injection they had been taken up by the pancreas and lung cells, whereas certain large vessels and the kidneys showed a lower abundance of such EXs. Moreover, the liver and gut cells were for the most part negative. These data thus suggest some target cell selectivity among EXs that are secreted *in vivo*.

Interestingly, EXs engineered to express a chimeric tetraspanin-8 in which the N-terminal region was swapped for a domain from the CD9 protein were readily taken-up by kidney cells but not by other organs, whereas EXs engineered to contain tetraspanin-8 that was fused to an integrin β4 was targeted preferentially to lung, kidney and gut cells. It is significant that also within an organ, EXs seem to be taken up preferentially by specific cell types depending on the exosomal protein content. One indication of this is the fact that tetraspanin-8; integrin β+ fusion EX products could be identified in only the kidney glomeruli (Rana et al., 2012).

There are several reports stating that EXs produced by kidney tumor cells are coupled to cancer-associated immune suppression (Yang et al., 2013; Diao et al., 2015; Gu et al., 2015). As an example of this, EXs purified from human kidney adenocarcinoma ACHN cells inhibited proliferation and induced apoptosis of Jurkat-immortalized T-cells while reducing *in vitro* interleukin-2 (IL-2), IL-6, IL-10 and interferon-γ production (Yang et al., 2013). In the light of these findings, the authors proposed that the Fas ligand within the tumor-derived EXs must be responsible for exosomally induced T-cell death.

There is considerable evidence that EXs play a role in renal tumor progression *in vivo*. The survival of mice inoculated with renal adenocarcinoma Renca cells, which go on to generate tumors, was reduced to some degree in the presence of Renca-derived EXs in the assay (Gu et al., 2015), and similar data have been reported by Diao et al. (2015), leading them to propose that the heat shock protein HSP70 present in EXs derived from cancer cells promotes the immunosuppressive activity of myeloid-derived suppressor cells (MDSCs), possibly via an increase in Stat3, a signal transducer and activator of transcription 3-phosphorylation.

Besides suppressing the immune system, EXs can also do exactly the opposite. Certain tumor-derived EXs induce an immune response. This suggests that EXs may even offer opportunities for developing individualized tumor immunotherapies (Greening et al., 2015). Given the fact that tumor-derived EXs also contain immunosuppressive molecules that reduce their immunogenicity, it is important to learn in detail how these properties are regulated, opening new avenues for valuable therapeutics. Along these lines, EXs that are secreted by IL-12-expressing RC-2 human renal cancer cells exhibited greater anti-tumor effects than EXs derived from “wild type” renal cells or cytocine-supplemented IL-12 alone (Zhang et al., 2010). Such IL-12-containing EXs efficiently induced cell proliferation, the release of interferon-gamma and the specific cytotoxic effects of T-cells derived from cultured human peripheral blood cells.

It is also of interest that the mice that had tumors derived either from the mouse myeloid leukemia line WEHI3B or renal carcinoma Renca cells survived longer if they had been vaccinated beforehand with the EX-loaded dendritic cells from the tumor (Gu et al., 2015). In other words, the tumor cell-derived EXs seemed to be superior to the tumor lysates as a source of antigen. Interestingly, the immunosuppressive features of EXs do not detract from their capacity to serve as an antigen source for the dendritic cells (Gu et al., 2015). These properties make the EXs very promising components for the development of novel cancer therapies.

### Kidney/urinary exosomes as diagnostic biomarkers and therapeutic agents

EXs have a great potential for use as valuable diagnostic biomarkers, especially in the case of monitoring kidney malfunction. Since, many reviews have been published on the analysis of urinary EXs and their potential as diagnostic markers for kidney disease, injury, and transplant rejection, this topic will be covered in the present review only briefly (Knepper and Pisitkun, 2007; Dimov et al., 2009; Dear et al., 2013; Properzi et al., 2013; Musante et al., 2014; Ranghino et al., 2014; Salih et al., 2014).

As discussed, the urinary EX components can be assigned to specific nephron segments, the glomerulus, the proximal/distal tubule, Henle's loop and the collecting duct (Table 1; Pisitkun et al., 2004; Miranda et al., 2010; Dear et al., 2013; Musante et al., 2014). Many of these proteins can be associated with certain diseases, but they are not all necessarily linked directly to the kidney. The level of GPRC5B in the urinary EXs correlates with AKI, so that its values are elevated in cases of AKI by comparison with normal healthy subjects (Kwon et al., 2014), making GPRC5B a candidate diagnostic marker for AKI. Furthermore, levels of Fetuin-A were also found to be elevated in patients suffering from AKI (Zhou et al., 2006).

The review by Ranghino et al. (2014) summarizes several suitable urinary exosomal biomarkers for glomerular and tubular damage, including Wilms Tumor 1 Homolog (WT1), ATF3, and Neutrophil Gelatinase-Associated Lipocalin (NGAL). WT1 was found in urinary exosomes of patients who suffer from focal segmental glomerulosclerosis (FSGS; Zhou et al., 2008, 2013b) and in most diabetic patients (Kalani et al., 2013). ATF3 is another marker for AKI alongside Fetuin A and GPRC5B (Zhou et al., 2008). NGAL levels were elevated in patients with delayed graft function after kidney transplantation (Alvarez et al., 2013), and several protein markers have also been found in cases of diabetic nephropathy (Zubiri et al., 2014) and prostate (Lu et al., 2009; Mitchell et al., 2009) and urine bladder cancer (Smalley et al., 2008; Blackwell et al., 2014).

Some markers that classify a person as healthy have also been identified, and these may be of diagnostic value in cases of kidney regeneration. Promonin-1 (CD133) serves as such a marker, for example, as it is lost in the urine at the end stage of renal disease (Dimuccio et al., 2014), and additional markers have been defined for a panel of kidney diseases that include diabetic nephropathy (Musante et al., 2014), cardio-renal syndrome (Gonzalez-Calero et al., 2014), autosomal-dominant polycystic kidney disease (ADPKD; Fang et al., 2013; Ben-Dov et al., 2014) and Gitelman's and Bartter syndromes (Corbetta et al., 2015), as well as for following the organ-acceptance after a kidney transplantation (Alvarez et al., 2013).

In 2007 the group led by Jan Löttvall found that EXs carry different functional RNA species (Valadi et al., 2007). Renal mRNA levels have been used in the past as prognostic markers for kidney diseases (Eikmans et al., 2003), but this required an invasive kidney biopsy. Analysis of the protein and RNA content of urinary EXs provides a non-invasive alternative for evaluating changes in renal gene and protein expression, and it has been found that several exosomal microRNAs isolated from urine are suitable markers for certain kidney diseases. One study showed that exosomal miR-145 and miR-130a levels were elevated in patients with diabetic nephropathy, while levels of miR-155 and miR-424 were down-regulated (Barutta et al., 2013). Further studies with a larger group of patients would be necessary to confirm these results.

Several miRNA markers of CKD/renal fibrosis have been identified. Levels of exosomal miR-29a, miR-29c, miR-200b, and miR-200c were down-regulated in patients with moderate-to-severe fibrosis (Lv et al., 2013), but not in cases with mild fibrosis. The same paper also demonstrates that miR-29c provides indicators of renal function and the histological degree of fibrosis, making it the most prominent candidate for a biomarker of CKD, while (Lv et al., 2014) showed that the exosomal mRNA level of CD2-associated protein (CD2AP) was down-regulated in CKD patients, and even more so in patients with a more severe disease. Both of these reports not only identified disease markers, but also demonstrated that the level of miRNA/mRNA present in urinary EXs provides an opportunity to define the progression of the disease.

Protocols for analyzing exosomes and isolating RNA from them still have to be optimized further in order to yield unbiased, reliable results. Furthermore, it still needs to be ascertained whether levels of exosomal mRNA can also reflect levels of the proteins used as biomarkers for kidney diseases. The same RNA and protein markers as were used in the past when a kidney biopsy was performed might not apply to exosomal RNA and protein levels, as is supported by the fact that little equivalence was found between the exosomal and cellular RNAs of exosome-producing cells (Skog et al., 2008; Mittelbrunn et al., 2011; Koppers-Lalic et al., 2014). This implies an underlying mechanism for the targeted loading of certain RNA species into EXs, in contrast to the loading of the most abundant cellular RNA into EXs in order to discard it. Future studies will have to elucidate the sorting mechanisms responsible for this process. Nonetheless, it is this fact that makes EXs especially interesting, as mRNAs and miRNAs can influence gene expression in the recipient cell and providing the EXs with their therapeutic potential.

One therapeutic strategy would be that RNA-bearing EXs would deliver their cargo to specific malfunctioning target cells and could restore damaged or deregulated protein production. Several studies have shown that exosomes shuttle functional miRNA and influence the gene expression levels of target cells (Pegtel et al., 2010; Montecalvo et al., 2012; Chen et al., 2014c).

Another promising feature of EXs is to serve as a non-cytotoxic drug delivery system. The challenge still lies in loading the drugs onto the EXs without imperiling their biological properties (Suntres et al., 2013). Different methods for loading a defined cargo onto EXs have been established, as recently reviewed (Johnsen et al., 2014). This is a possible way of developing novel therapeutics to treat various diseases. Some examples exist of studies which are currently in clinical trial phase I.

One such investigation concerns the potential application of EXs to deliver curcumin (which has proved to have biological activity) to colon cancer tissue (clinical trial no. NCT01294072), while another uses EXs as a vaccine, administering dendritic cell-derived EXs (CSET 1437) loaded with antigen to lung cancer patients in order to activate their innate and adaptive immunity during therapy (clinical trial no. NCT01159288). Furthermore, the potential application of EXs to the treatment of kidney diseases is being explored. As described in Section The Role of Exosomes in Kidney Regeneration and Diseases above, various groups have shown that MSCs have a paracrine effect on acute and chronic kidney diseases (Gatti et al., 2011; van Balkom et al., 2011; Fang et al., 2013) in which soluble factors such as those associated with the secreted vesicles have a positive influence on cell behavior and promote the initiation of recovery. The potential of EXs for use in cell-free therapy applications has been summarized recently (Vishnubhatla et al., 2014) and will not be discussed here in detail. It is clear, however, that EXs from various cells such as MSCs and cancer cells have a great potential as novel therapeutic tools, and they can be expected to have a significant impact on the development of diagnostics and new treatments, for kidney diseases among others.

## Conclusions

It has become generally accepted for the moment that EXs are present in a wealth of body fluids and are not only a cellular waste system as was thought earlier. Collectively, they may represent a newly identified but apparently ancient humoral system controlling homeostasis and disease, and they also provide a useful bank of biomarkers for a variety of diseases and may raise the value of urine as a non-invasive diagnostic component in medical practice. The analysis of urinary EXs not only provides us with prognostic disease markers, but might also make it possible to differentiate between diseases which display similar symptoms. Furthermore, certain data have shown that it is possible to estimate the severity of a disease, and hence its progression. This could enable non-invasive monitoring of responses to treatment and also make it possible to look for complications after kidney transplantations. The use of EXs as therapeutics and vaccines seems to be safe and effective due to their target specificity and their lack of cytotoxicity. This and the fact that a specific cargo can be loaded into them also make them very promising candidates as novel drug delivery systems. This is still something of a challenge, however, and better protocols need to be developed. More research will be necessary to ensure the safety of the resulting medical applications.

**Figure 2:**
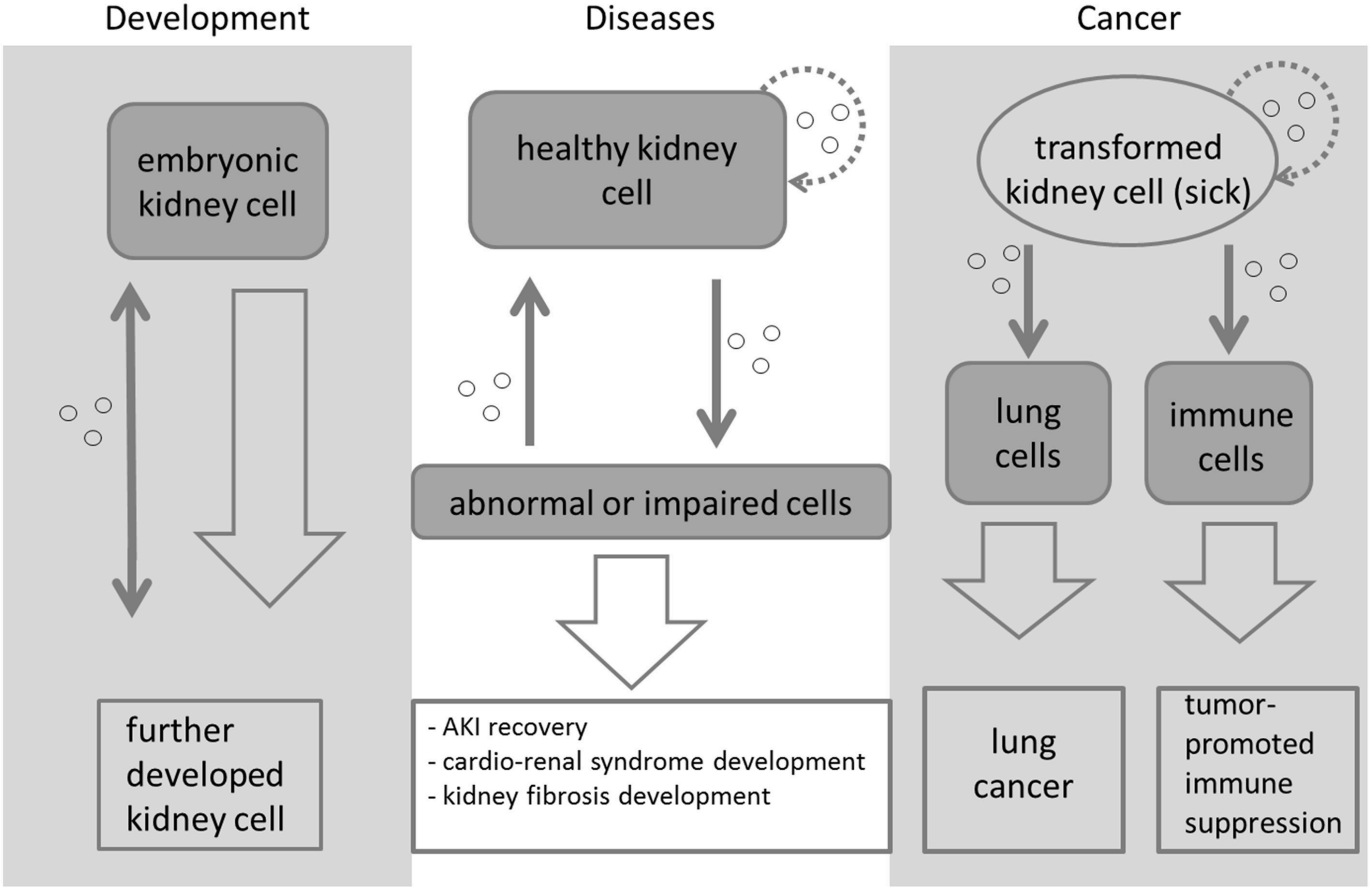
**Exosome-mediated signaling during kidney development, disease, and cancer**. The signaling is symbolized by a thin gray arrow, while the development from one cell type/cell state to another is symbolized with a larger arrow. Different types of embryonic kidney cells release vesicles (small circles) to transfer signals during morphogenesis. These initiate and regulate the development of the organ. Exosomes are a means of cell-to-cell communication. When abnormal kidney conditions appear, exosomes can be released from the abnormal cells to be subsequently taken up by healthy cells, which will then be transformed and might even become abnormal as well. It has been reported that exosomes released from transformed kidney cells can initiate cancer in lung cells (Grange et al., 2011; Rana et al., 2012) and lead to tumor-promoted immune suppression in certain immune cells (Yang et al., 2013; Diao et al., 2015; Gu et al., 2015). Kidney cells and transformed kidney cells also take up the released vesicles again (dotted lined arrows). Most of the means of signaling depicted here are still not very well-characterized or understood.

A schematic overview of the signaling role of EXs in relation to the kidney is presented in Figure 2. It is known that EXs play a role in kidney diseases and renal cancer, but many of the signaling molecules found during nephrogenesis occur in association with EXs, leading us to conclude that they not only participate in disease processes but also play a putative role in developmental processes. Which machinery regulates these processes during embryonic kidney development still remains largely an open question, but the accumulating evidence is starting to point toward a critical role for EXs in inductive signaling in the kidney. Future work should be targeted toward elucidating the normal physiological roles of EXs *in vivo* in defined disease models and advancing our knowledge of the precise mechanisms by which they promote renal diseases and cancer.

## Author contributions

MK drafted the manuscript and prepared the figures and tables. AS helped with writing the manuscript and designing the figures. AS and SV critically reviewed and revised the manuscript. All three authors read and approved final version of the manuscript.

## Funding

This work was supported by the Academy of Finland (206038, 121647, 250900, and 260056), Centre of Excellence grant 2012–2017 of the Academy of Finland (251314), the Sigrid Jusélius Foundation and the European Community's Seventh Framework Program, FP7/2009, under grant agreement 305608 (EURenOmics: European Consortium for High-Throughput Research in Rare Kidney Diseases).

### Conflict of interest statement

The authors declare that the research was conducted in the absence of any commercial or financial relationships that could be construed as a potential conflict of interest.
